# The Mechanical Benefit of Medial Support Screws in Locking Plating of Proximal Humerus Fractures

**DOI:** 10.1371/journal.pone.0103297

**Published:** 2014-08-01

**Authors:** Wen Zhang, Langqing Zeng, Yanjie Liu, Yao Pan, Wei Zhang, Changqing Zhang, Bingfang Zeng, Yunfeng Chen

**Affiliations:** 1 Department of Orthopaedic Surgery, Shanghai Jiao Tong University Affiliated Sixth People's Hospital, Shanghai Jiao Tong University, Shanghai, China; 2 Department of Orthopaedics, Zhuhai People's Hospital, Jinan University Affiliated Zhuhai Hospital, Guangdong, China; University of Eastern Finland, Finland

## Abstract

**Background:**

The purpose of this study was to evaluate the biomechanical advantages of medial support screws (MSSs) in the locking proximal humeral plate for treating proximal humerus fractures.

**Methods:**

Thirty synthetic left humeri were randomly divided into 3 subgroups to establish two-part surgical neck fracture models of proximal humerus. All fractures were fixed with a locking proximal humerus plate. Group A was fixed with medial cortical support and no MSSs; Group B was fixed with 3 MSSs but without medial cortical support; Group C was fixed with neither medial cortical support nor MSSs. Axial compression, torsional stiffness, shear stiffness, and failure tests were performed.

**Results:**

Constructs with medial support from cortical bone showed statistically higher axial and shear stiffness than other subgroups examined (P<0.0001). When the proximal humerus was not supported by medial cortical bone, locking plating with medial support screws exhibited higher axial and torsional stiffness than locking plating without medial support screws (P≤0.0207). Specimens with medial cortical bone failed primarily by fracture of the humeral shaft or humeral head. Specimens without medial cortical bone support failed primarily by significant plate bending at the fracture site followed by humeral head collapse or humeral head fracture.

**Conclusions:**

Anatomic reduction with medial cortical support was the stiffest construct after a simulated two-part fracture. Significant biomechanical benefits of MSSs in locking plating of proximal humerus fractures were identified. The reconstruction of the medial column support for proximal humerus fractures helps to enhance mechanical stability of the humeral head and prevent implant failure.

## Introduction

Proximal humerus fractures account for approximately 5% of all fractures [1]–[3]. Surgical intervention is generally accepted for unstable fractures, including displaced fractures and fractures associated with osteoporosis. Recent research noted a high failure rate(8.6%–22.0%) after open reduction and internal fixation (ORIF) of proximal humerus fractures [4]–[7]. Fixation without the reconstruction of medial support is considered one of the risk factors of implant failure [5]. Gardner et al. demonstrated the direct association between medial support and subsequent loss of reduction [8]. Zhang et al. found that insertion of a medial support screw (MSS) into the medio-inferior region increased the stability of fixation of complex fractures and reduced the risk of implant failure[9]. When medial comminution and malreduction are present at the proximal humerus, the insertion of a MSS precisely to the medio-inferior region of the humeral head is one method to reconstruct the medial column support.

Investigation into the value of MSSs in the treatment of proximal humerus fractures has been limited to clinical outcome studies. No biomechanical studies have been reported. The purpose of this study was to evaluate the biomechanical advantages of MSSs in the locking plate for the treatment of proximal humerus fractures and to use this information to guide clinical practice.

## Methods

**Figure 1:**
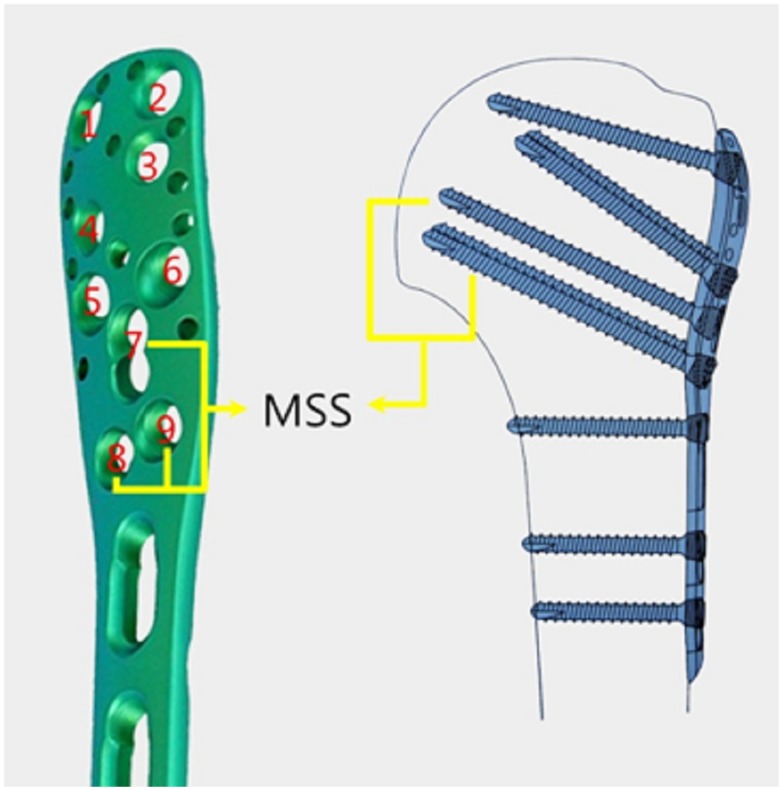
The proximal screw distribution and the medial support screws (MSS) for the locking proximal humerus plate.

A total of 30 adult synthetic left humeri (HI-C type, Orthobone, Hangzhou, China) and 30 sets of proximal humerus locking plates and screws (Double medical, Xiamen, China) (Fig. 1) were prepared. All the mechanical tests were performed on an Axial-Torsional Biomechanical Testing System (301.6 Shore Western Manufacturing, California,USA) with the following load cell characteristics: a maximum force rating of 3,200 pounds (14.2 kN) and a stroke of four inches (100 mm). The torsional rating on this unit is 1,000 inch-pounds (120 Nm) with a rotary motion capability of ±140°.

The 30 synthetic humeri were randomly divided into three groups (*ie*, Groups A, B, and C with 10 specimens/group). A two-part surgical neck fracture was created in each proximal humerus, and all fractures were fixed with a locking proximal humerus plate.

### Fracture groups

**Figure 2:**
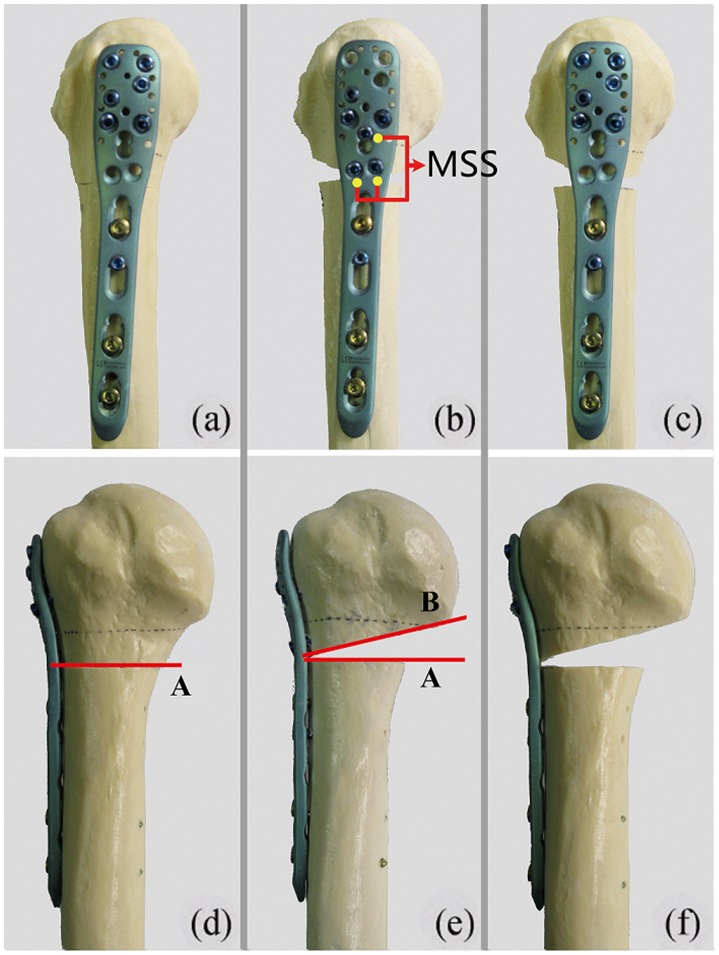
Division of the proximal humerus fracture models. In group A, proximal humerus fractures were fixed without MSSs (Fig.2a); transverse osteotomies were created along a horizontal line (line A) (Fig. 2d). In group B, proximal humerus fractures were fixed with 3MSSs(Fig.2b); wedge osteotomies were created along a horizontal line(line A) and an oblique line (line B) to simulate medial comminution of the proximal humerus(Fig. 2e). In group C, proximal humerus fractures were fixed without medial cortical support or MSSs(Fig.2c); wedge osteotomies were created identical to group B (Fig. 2f).

Group A (Fig. 2a and Fig. 2d). Ten proximal humerus fractures were fixed with medial cortical support; however, MSSs were not used. A horizontal line (line A) was made 1 cm distal to the humeral head, and transverse osteotomies were created along this line using an industrial bandsaw to simulate a two-part surgical neck fracture of the proximal humerus. Fractures were anatomically reduced and fixed with a locking proximal humerus plate. Six locking screws were inserted into the proximal holes of the plate from No. 1 to No. 6. (Fig 1).

Group B (Fig. 2b and Fig. 2e). Ten proximal humerus fractures were fixed with 3 MSSs; however, no medial cortical support was provided. A horizontal line (line A) was made 1 cm distal to the humeral head. The intersection of line A and the lateral cortex of the proximal humeral metaphysis was identified, and a line (line B) was drawn from this point to the most medio-inferior point of the humeral head. Wedge osteotomies were created along lines A and B to simulate medial comminution of the proximal humerus. Three locking screws were inserted through holes No. 7–9(Fig 1) into the medio-inferior region of the humeral head at the proximal part of the plate. Another three screws were randomly inserted into the other six holes (No. 1–6) of the proximal part of the plate.

Group C (Fig. 2c and Fig. 2f). Ten proximal humerus fractures were fixed without medial cortical support or MSSs. The fracture model was constructed identical to Group B. However, six locking screws were inserted into holes No. 1 to 6 of the proximal part of the plate.

The proximal screws in all three groups were inserted 5 to 8 mm below the subchondral bone to simulate clinical practice [10]. Humeral shafts were fixed with one cortical screw and three locking screws. Plate-bone gap was not present in all experimental models. The distal part of the humeri were removed 15 cm distal to line A. Specimens were 20 cm long, and the distal portion of each specimen was fixed by a square steel chamber filled with a commercially-available anchoring cement to a depth of 12 cm.

### Biomechanical testing

#### Axial stiffness

**Figure 3:**
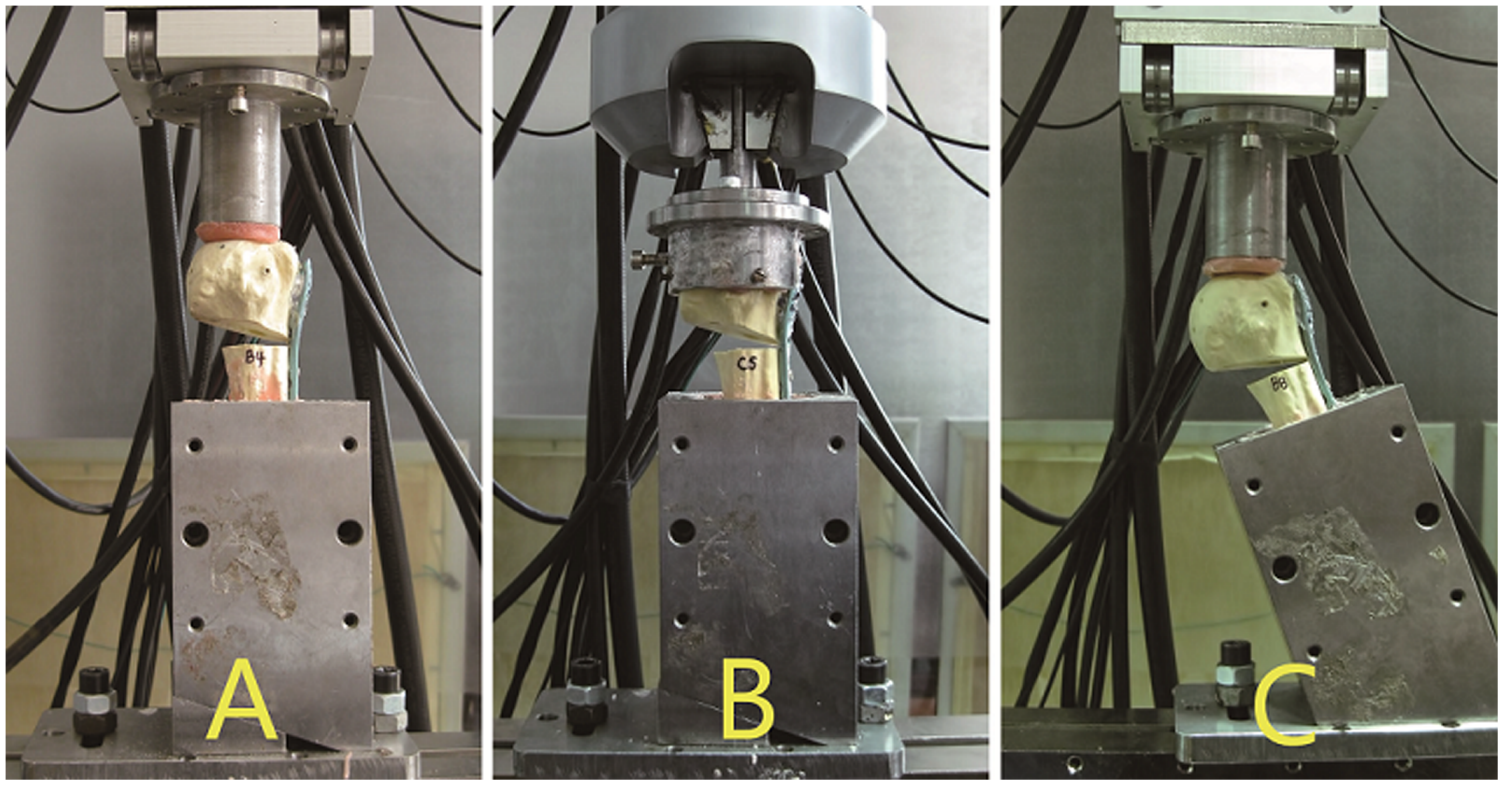
Mechanical test modes used to assess the (A) axial stiffness; (B) torsional stiffness; and (C) shear stiffness and load-to-failure of the plated humeral constructs.

Each humerus was oriented vertically in the coronal and sagittal planes. Using a plate attached to the mechanical tester, axial compression was applied with a vertical load at the apex of the humeral head using displacement control (max deflection = 0.5 mm; load rate = 5 mm/min [11]; preload = 50 N). The maximum load was recorded, and the slope of the load-deflection curve was used to compute the axial baseline stiffness. Each test was repeated three times, and the average maximum load and average stiffness were calculated (Fig. 3a). Every specimen was kept within the linear elastic region to prevent any permanent damage (average linearity coefficient R^2^>0.99). No visual evidence of damage was noted.

#### Torsional stiffness

Each humerus was positioned inside the cup of a cylindrical stainless steel block and secured using three 4.0 mm screws that were inserted into the humeral head. A torque was applied using displacement control (maximum angulation = 5°; rate = 12°/min; pre-torque = 0 Nm) to simulate rotation of the humeral head. The maximum torque was recorded, and the slope of the torque-angulation graph was used to determine torsional stiffness. The application of torque was repeated three times, and an average maximum torque and average torsional stiffness were calculated (Fig. 3b). All specimens were kept within the linear elastic region to avoid permanent specimen damage (average linearity coefficient R^2^>0.99). No visual evidence of damage was noted.

#### Shear stiffness

Each humerus was mounted distally in a vice with the shaft axis in 20° of abduction(Fig.3c) as recommended by Koval et al. in order to simulate the shear loading across a proximal fracture site experienced when rising out of a chair or crutch weight bearing [11].The same test procedure as that with axial stiffness testing was performed, except that maximum displacement was 1.0 mm. Load levels were chosen to prevent permanent damage to the specimens. All specimens remained within the linear elastic region to avoid permanent damage (average linearity coefficient R^2^>0.99). No visual damage was noted.

#### Strength testing

Load-to–failure in shear for each of the three groups was determined using displacement control (load rate = 5 mm/min; preload = 50 N) to apply a vertical force to generate shear compression on each specimen oriented 20° in abduction (Fig.3c). The force was applied until catastrophic failure of the implant occurred. The highest peak load was determined to indicate significant structural collapse [11]. Catastrophic fracture patterns of the bone and implant were examined and recorded.

### Statistical analysis

For the statistical analysis, the SAS 11.0 (SAS Institute Inc, North Carolina, USA) was used. Statistical analyses were performed by an independent statistician blinded to surgical outcomes. The Student's t-test was employed with the one-way analysis of variance (ANOVA) test used for continuous variables. The Turkey *post hoc* test was used to differentiate groups for statistical differences. The level of statistical significance was set to *P*<0.05. The chi-square test or the Fisher's exact test was employed for binary variables with the level of statistical significance set to *P*<0.05.

## Results

There was no evidence of plate or screw loosening or breakage. No fractures occurred during axial, torsional and shear stiffness tests. The results of four different load tests for the three groups are shown in Table 1.

**Table 1:** The results under four different load tests for the three groups (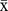
±sd, n = 10).

Group	Torsional stiffness test (max angulation = 5°)		Axial stiffness test (max displacement = 0.5 mm)		Shear stiffness test (max displacement = 1 mm)		Shear failure test
	Maxtorque (NM)	Torsional stiffness (NM/deg)	Max load (N)	Axial stiffness (N/mm)	Max load (N)	Shear stiffness (N/mm)	Shear failure load (N)
Group A	8.92±0.25	1.80±0.07	240.9±19.1	424.4±101.2	444.7±20.9	470.0±54.4	2949.8±355.1
Group B	9.09±0.31	1.86±0.07	169.0±19.3^a^	230.7±40.54^a^	228.8±29.0^a^	183.9±29.6^a^	2448.1±402.4^a^
Group C	7.57±0.53^a^^,^ b	1.53±0.10^a^^,^ b	128.6±17.5^a^^,^ b	147.0±29.2^a^^,^ b	188.7±26.2^a^^,^ b	140.2±32.1^a^	2222.6±336.4^a^
*F* value	47.06	45.14	92.94	47.67	290.53	198.05	10.36
*P* value	<.0001	<.0001	<.0001	<.0001	<.0001	<.0001	0.0005
Comparison	A vs B: 0.6086	A vs B: 0.2738	A vs B: <0.001	A vs B: <0.001	A vs B: <0.001	A vs B: <0.001	A vs B: 0.0131
of three	A vs C: <0.001	A vs C: <0.001	A vs C: <0.001	A vs C: <0.001	A vs C: <0.001	A vs C: <0.001	A vs C: 0.0004
group	B vs C: <0.001	B vs C: <0.001	B vs C: <0.001	B vs C: 0.0207	B vs C: 0.0044	B vs C: 0.0561	B vs C: 0.3655

a compared with group A, P<0.05;

b compared with group B, *P*<0.05.

### Axial stiffness

**Figure 4:**
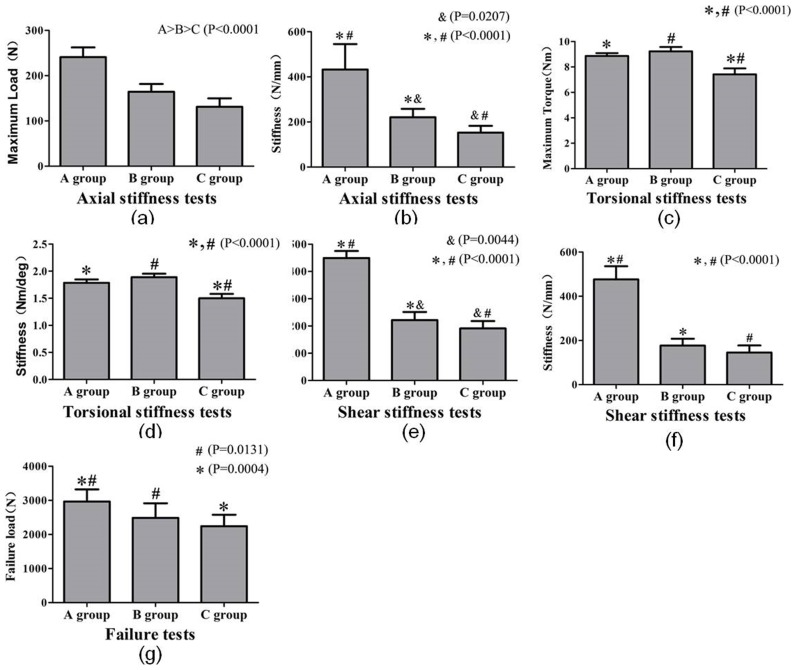
Comparison of stiffness tests among three groups under four load steps (a,b) axial stiffness test, (c, d) torsional stiffness test, (e, f) shear stiffness test, (g) failure test. Fig(Group A> Group B> Group C; *P*≤0.0207) Fig 4 c Torsional stiffness data for all subgroups. The maximum load of Group C was statistically different from both Group A and Group B, (Group A> Group C, Group B> Group C; *,#, *P*<0.0001). The comparisons of Groups A and B for maximum load were not significantly different (*P* = 0.6086). Fig 4 d Torsional stiffness data for all subgroups. The torsional stiffness of Group C was statistically different from both Group A and Group B (Group A> Group C, Group B> Group C; *,#, *P*<0.0001). The comparisons of Groups A and B for torsional stiffness were not significantly different (*P* = 0.2738). Fig 4 e Shear stiffness data for all subgroups. The maximum load showed statistical differences between the groups (Group A> Group B> Group C; *P*≤0.0044). Fig4 f Shear stiffness data for all subgroups. The shear stiffness of Group A was statistically different from both Group B and Group C (Group A> Group B, Group A> Group C; *,#, *P*<0.0001). The comparisons of Groups B and C for shear stiffness were not significantly different (*P* = 0.0561). Fig 4 g The load-to-failure of Group A was statistically different from both Group B and Group C (Group A> Group B, Group A> Group C; *,#, *P*≤0.0131); however, no statistical differences were noted between Group B and Group C (*P* = 0.3655).

The maximum load (max displacement = 0.5 mm) and the axial stiffness showed statistical differences between the groups (Group A> Group B> Group C; P≤0.0207) (Fig. 4a, Fig 4b).

The maximum loads were 240.88 N±19.13 in Group A, 169.04 N±19.26 in Group B, and 128.58 N±17.53 in Group C. The axial stiffness was 424.4 N/mm ±101.2 for Group A, 230.7 N/mm ±40.54 for Group B, and 147.0 N/mm ±29.2 for Group C.

### Torsional stiffness

The maximum load of Group C (7.57 Nm ±0.53) was statistically different from both Group A (8.92 Nm ±0.25) and Group B (9.09 Nm ±0.31) (Group A> Group C, Group B> Group C; *P*<0.0001). The torsional stiffness of Group C (1.53 Nm/deg ±0.10) was statistically different from both Group A (1.80 Nm/deg ±0.07) and Group B (1.86 Nm/deg ±0.07) (Group A> Group C, Group B> Group C; *P*<0.0001); however, the comparisons of Groups A and B for maximum load (*P* = 0.6086) and torsional stiffness (*P* = 0.2738) were not significantly different (Fig 4c, Fig 4d).

### Shear stiffness

The maximum load between subgroups showed statistical differences (Group A> Group B> Group C; *P*≤0.0044)(Fig 4e). The values were 444.7 N ±20.9 for Group A, 228.8 N±29.0 for Group B, and 188.7 N±26.2 for Group C.

The shear stiffness of Group A(470.0 N/mm ±54.4) was statistically different from both Group B(183.9 N/mm ±29.6) and Group C(140.2 N/mm ±32.1)(Group A>Group B, Group A>Group C; P<0.001); however, the comparisons of Group B and Group C for shear stiffness were not significantly different(P = 0.056). (Fig 4f).

### Shear failure

The load-to-failure of Group A (2949.76 N±355.08) was statistically different from both Group B (2448.13 N±402.39) and Group C (2222.55 N±336.41) (Group A> Group B, Group A > Group C; *P*≤0.0131); however, no statistical differences were noted between Group B and Group C (*P* = 0.3655). (Fig. 4g).

### Shear failure mode

**Figure 5:**
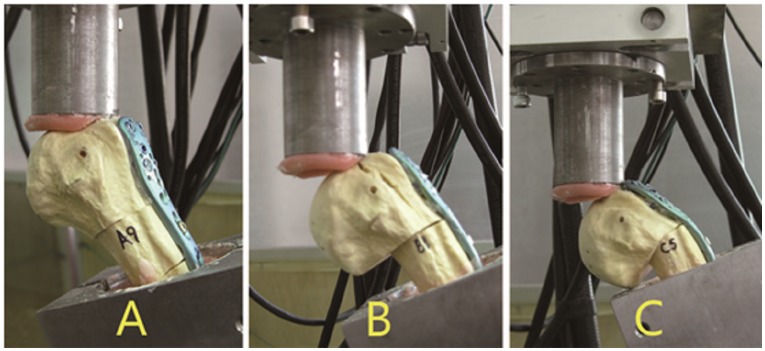
Shear Failure Mode: humeral shaft fracture (A), humeral head fracture (B) and specimens failed by significant plate bending (C).

In group A, seven specimens failed by humeral shaft fracture (Fig. 5A), and three specimens failed by humeral head fracture. No plate bending occurred in Group A. In group B, five specimens failed by plate bending with humeral head collapse, and five specimens failed by humeral head fracture (Fig. 5B). In group C, six specimens failed by significant plate bending with humeral head collapse (Fig. 5C), and four specimens failed by humeral head fracture.

## Discussion

Biomechanical tests are currently being used in studies of fracture fixation [11]–[14]. In the current study, biomechanical tests showed that medial support from cortical bone in the proximal humerus provided the best stability when locking plates were used to treat proximal humerus fractures. When the proximal humerus was not supported by medial cortical bone, locking plating with MSSs exhibited higher biomechanical performance than locking plating without MSSs. There were differences among the three groups in the mode of failure. When the proximal humerus was supported by medial cortical bone, specimens showed no evidence of plate bending and failed primarily by fracture of the humeral shaft or humeral head. When the proximal humerus was not supported by medial cortical bone, specimens failed primarily by significant plate bending at the fracture site during axial loading. The humeral head was then crushed by the medial cortical bone of the humeral shaft, which caused a humeral head fracture.

A recent biomechanical study by Lescheid et al. also demonstrated that locking plating with medial cortical support was more resistant to axial compression and shear force compared with locking plating without medial cortical support [12]. However, there was no significant difference between specimens with medial cortical contact and other subgroups, which was different to our results. This difference may be resulted from the small number of specimens included in their research, only 6–7 specimens were included in each subgroup. In addition, the supporting role of MSS was not studied in their research. The effect of the MSS was limited to the clinical literature with no evidence of biomechanical research. Our study was conducted to verify the biomechanical benefits of the MSSs in proximal humerus locking plates and to use this information to guide clinical practice.

Based on our results, strong medial support should be reconstructed in every case. When treating two-part proximal humerus fractures, anatomical reduction of the medial cortical bone should be obtained. When the medial cortical bone is comminuted or cannot be anatomiacally reduced, 2 or 3 MSSs can be inserted in order to help the reconstruction of the medial support. If medial support is not reconstructed, the resistance to axial compression and torsional force decreases, and early failure of the fracture fixation can occur.

Compared with traditional fixation methods, the fixed-angle devices can provide a greater ability to resist angular and rotational forces, especially in osteoporotic patients [15], [16]. Friess et al. also found that locking plates showed higher performance on both the functional range of motion and American Shoulder and Elbow Surgeons (ASES) scores after a mean 45-month follow-up [16]. However, locking plating still has early failures, especially in patients with comminuted and osteoportic fractures or fixation without reconstruction of medial support [5], [6]. Common complications include varus deformity (16%), humeral head necrosis (10%), and screw cut out (8%) [17]. Fixation without reconstruction of medial support is one of the risk factors for implant failure [5]. Reconstruction of the medial support increases the stability of fixation by providing effective support to the humeral head [8], sharing the varus deforming force, and decreasing the cutting force between the screws and the bone. Therefore, it is advisable to reconstruct the medial support of proximal humerus during the operation.

Anatomical reduction of the medial cortical bone of the proximal humerus is one of the methods to reconstruct the medial column support of the proximal humerus [8]. The current study confirmed that the medial cortical bone support of the proximal humerus has the best biomechanical stability. When the medial cortical bone of the proximal humerus is anatomically reduced, the cortical bone contacts, and the supporting forces are created to increase axial, torsional, and shear stiffness. Therefore, for a simple fracture of the proximal humerus, attempts should be made to achieve anatomical reduction of the medial cortical bone to gain medial column support and help avoid implant failure. When the medial metaphysis of the proximal humerus is comminuted, fractured with a bony defect, or the fracture is malreduced, one or two additional locking screws can be inserted obliquely into the medio-inferior region of the humeral head to reconstruct medial column support of the proximal humerus [10]. A cadaveric biomechanical study by Liew et al. found that the grasping force of a screw placed under the subchondral bone of the medial and inferior region was comparably stronger than that of a screw placed either in the middle of the humeral head or in the lateral and superior region[18]. Another histomorphometric study by Hepp et al. showed the highest bone strength to be in the medial and dorsal aspects of the proximal humeral head[19]. As a result, the optimal fixation of a screw is in the posterior-medial-inferior aspect of the humeral head to prevent screw cut out and implant loosening. Furthermore, MSSs in hole No. 7∼9 were comparably closer to the fracture line than that of a screw placed in hole No. 1∼6, and MSSs were relatively upwards inserted. These screws would support the proximal fragment directly from the medio-inferior part of the humeral head and might be conducive to the resistance to varus deforming force. Zhang et al. and Hardeman et al. found MSSs increase the stability of proximal humerus fractures [9], [20]. However, their studies were limited to clinical outcomes with no evidence of biomechanical study. Through biomechanical tests, our study proved when the medial cortical bone of the proximal humerus is fractured with comminution, MSSs could help to increase the stability of fixation and should be strongly considered.

Several methods exist for the reconstruction of medial column support in comminuted proximal humerus fractures with bony defects. Egol et al. performed fracture site augmentation with calcium phosphate cement to increase stability after ORIF of the proximal humerus fractures [21]. Micic et al. achieved anatomical reduction with autogenous bone graft into the area of medial comminution [22], while Hettrich et al. augmented the fixation with a fibular allograft inserted medially[23]. However, there is still no gold standard treatment for reconstruction of medial column support in comminuted proximal humerus fractures. And to some extent, the bone graft methods mentioned above need more stripping of soft tissue at the medial side of the proximal humerus, which is important for the blood supply of the humeral head. In our opinion, inserting two to three MSSs will help the reconstruction of the medial support if the supporting screws can be inserted precisely into the medio-inferior region of the humeral head. To achieve this, we need to adjust the locking plate to the appropriate height and confirm the position of the tips of the MSSs by intraoperative fluoroscopy. This procedure does not require stripping a wide range of soft tissue or excessive manipulation of the fracture fragments. For comminuted and osteoporotic fractures, tension band suture is routinely used as supplemental fixation to improve the stability of the humeral head[24], [25].

There are a number of limitations in this study. First, the effect of the surrounding soft tissues on the mechanical stability of the construct was not evaluated. Second, synthetic bone may more closely simulate normal, rather than osteoporotic, bone. The biomechanical characteristics were likely different from the complicated proximal humerus fractures seen in osteoporotic patients, especially the failure mode. More specimens might have failed by humeral head fracture or humeral head collapse if osteoporotic specimen were used. However, under the same objective conditions, the same experimental procedure was used to assess the mechanical stiffness and strength of each proximal humerus fracture fixation for three subgroups (medial cortical support, MSSs, and no medial column support). Third,the effect of cyclic loading was not investigated, which may be more predictive of the long-term performance of the construct than static load. Fourth, in order to make the study closer to clinical practice, we did not specify which three holes to be used for screw insertion in No. 1∼6 in group B. Clinically, the number of holes selected depends on the specific condition in the operation such as fracture configuration. While screw position of the aforementioned three holes might have impacted on our results, we are unable to quantify its contribution. Future studies using finite element method will be undertaken to further investigate this.

## Conclusions

Proximal humerus fracture fixation with medial cortical contact demonstrated the best biomechanical characteristics. Every effort should be made to achieve anatomical reduction of the medial cortical bone of the proximal humerus. Constructs with three MSSs showed statistically higher axial, torsional, and shear stiffness than constructs without medial support. Therefore, it is recommended that if the medial comminution is present with bony defect or the medial cortical bone is malreduced, three MSSs should be inserted to reconstruct medial column support of the proximal humerus to prevent postoperative implant failure.
